# Occupational Health and Quality of Life of Health Care Workers in the “Return to Work” Stage

**DOI:** 10.3390/healthcare11020204

**Published:** 2023-01-10

**Authors:** Maria Malliarou

**Affiliations:** Laboratory of Education and Research of Trauma Care and Patient Safety, Faculty of Nursing, University of Thessaly, 41110 Larisa, Greece; malliarou@uth.gr

Globally, over 59 million people are employed as health care workers [1] including generalist medical practitioners, specialist medical practitioners, nursing professionals, midwifery professionals, traditional and complementary medicine professionals, etc. [2]. Τhis diversity of specialization presented by hospitals has the effect of multiplying risks and consequently increasing the chances of accidents.

Hospitals are considered one of the most hazardous places to work, with more than 500,000 injuries annually. The likelihood of injury or illness, resulting in days away from work, is higher in hospitals than in construction and manufacturing [3]. Injuries accounted for 93 percent of the total cases of days away from work recorded; illnesses accounted for the remaining 7 percent. The most common injuries resulting in days away from work are musculoskeletal disorders and, more specifically, sprains and strains, which account for 54 percent of these injuries. The top six injury categories are bruises, soreness, fractures, multiple traumas, and cuts and punctures [3].

The psychology of the employees does not remain unaffected, and they often show signs of psychosocial fatigue. More specifically, these psychosocial consequences present as depressive symptoms, social isolation and inactivity, reduced well-being, impaired self-image, and high risk of disability pension [4,5,6,7,8,9].

In addition, the large rates of absence from work result in economic consequences, not only for the health of the employees, but for the organization as well [10]. After a 12-week work absence, the chance of an employee returning to work drops by 50% [11]. Thus, it is important to comprehend why some employees return to work, while others delay it, become permanently disabled, or prematurely exit the workforce. 

But what really happens when health care workers must return to work after an injury or illness? Can they be 100% functional? And if not, what measures should the organization take for them to return smoothly to normality without any further costs?

In conclusion, interventions aiming at helping health care employees returning to work might be beneficial. A creative, supportive, and challenging working environment can make employees more confident in returning after a long period of absence.
